# Fulton County Medical Society

**Published:** 1908-09

**Authors:** R. R. Daly


### FULTON COUNTY MEDICAL SOCIETY.

---

REGULAR MEETING, JULY 16, 1908, CARNEGIE LIBRARY, AT 8 P. M.
REPORTED BY R. R. DALY, M. D.

---

None of the essayists for the evening being present, the session was given over to informal discussion of cases.

*Dr. Daly* described a case of petit mal in a boy of eleven. The seizures were frequent, 10 to 20 a day—but lasted only a few seconds. During these he sometimes fell and lost consciousness. He was photographed falling on a mattress. Another young boy was associated with him several weeks and then he, too, began to have seizures. These were judged to be imitative. The lad was soundly spanked for mimicing his associate and the simulated seizure ceased.

Children should be protected from associating with epileptics.

*Dr. Olmstead* reported a case of epilepsy with periods of amnesia lasting from 2 to 48 hours after the attack. The man would lose consciousness on the street and go home without remembering any of his movements. Sometimes he would fall in major convulsion and he has been taken to Grady Hospital at times. The medicolegal importance of such cases impressed the speaker forcibly. A crime might easily be committed during this epileptoid condition for which the patient would be entirely irresponsible. Indeed this has occurred and a case was cited showing the unfortunate complications.

*Dr. Sellman* reported a case of peculiar eruption occurring in a young woman. It appeared first as a macule, quickly becoming papular and surrounded by red circle. It rapidly became confluent and beginning on the arms covered nearly the entire body except the face. It increased in color to a bright red. It came on, as near as he could judge, from the symptoms of malaise at the fifth day and lasted about a week. After the rash had entirely disappeared there was general desquamation of the skin. The patient recovered uneventfully.

Several doctors were in consultation with him and the diagnosis was variously made as typhoid fever, typhus fever, measles, syphilis and scarletina. The typhoid condition was not marked, but there was fluctuating temperature and prostration. The increasing eruption suggested typhus, though it was not at all characteristic. Confluent measles was set aside because of no catarrh and scarlatina was thought of chiefly by the manner of peeling at the close of the fever. No one suffered any contagion, though many saw the case.

Dr. Sellman closed by saying he did not yet know what the disease was.

*Dr. Hutchins* said that it probably was a case of pityriasis maculata et circinata. In this disease there is an early disquamation, slight in amount, that might have been overlooked.

Again it might have been one of those unclassified eruptions due to any toxaemia from which the patient may have been suffering.

*Dr. Thrash* continued to believe it was typhoid fever. He saw the case and formed his opinion then.

*Dr. Olmstead* said typhoid was not to be mistaken in such a case. He agreed with Dr. Hutchins in the toxic origin.

*Dr. Thrash* related a case of small abscess in the neck of a young patient. It was opened, but did not get well and the patient returned to him on that account. Several biologic examinations were made by Dr. Harris and others without any definite result. Rabbits were inoculated with the secretion and seemed to thrive. After all sorts of things had been tried including several serum injections, though tuberculin was not used on account of the good condition of the rabbits, the patient developed an acute milliary tuberculous of the lungs and died. No post-mortem was obtainable. Three and a half months after the first injection, the rabbits died of tuberculosis—too late to be of assistance in diagnosing the condition of the patient.

The slow, obscure progress of the disease was remarkable and confused all those who examined it.

*Dr. Armstrong* reported a case of ruptured appendix in which he did a drainage operation as a last chance and expected the patient to die. He placed him in the semi-sitting posture—the Fowler position—and a recovery followed. He felt the man was too weak for constant rectal enemata or he would have used that means also. The Fowler position not only increased drainage, but it kept the secretions in the pelvic part of the peritoneum which is less absorbent than the diaphragmatic.

*Dr. Hodgson* described a case of small tumor of the breast that had been growing at least four years. It was loose in all directions and he prepared for a simple enucleation under the breast. He found the capsule ruptured and the outspreading part showing enough of malignancy to call for a radical operation, which he then performed. Later examination showed progressive changes in the tumor even to the giant cells in the mushroom like process outside the capsule. The case gives additional warrant for the early operation upon all breast tumors.

*Dr. Stirling* reported a case showing difficulty in diagnosis in mastoid troubles. There had been recurrent furunculosis in the external meatus and periostitis as well, but these painful points had subsided under treatment. There was no discharge from the tympanum nor any evidence of trouble there.

After several weeks the patient returned with marked pain and tenderness over the mastoid and was operated upon. There was extensive disease all through the mastoid requiring general currettage, but the tympanum was not involved in any way. It is these obscure cases doubtless that cause much of the meningitis the family doctor cannot account for.

### PRESIDENT ROOSEVELT HAS ACCEPTED THE PRESIDENCY OF THE INTERNATIONAL CONGRESS ON TUBERCULOSIS. HIS LETTER TO DR. LAWRENCE F. FLICK, CHAIRMAN OF THE COMMITTEE OF ARRANGEMENTS FOR THE CONGRESS.

---

THE WHITE HOUSE.
Washington, May 12, 1908.

Sir: It is with great pleasure that I accept the presidency of the "International Congress on Tuberculosis" which is to meet in this city on September 21, 1908, and extend its session to October 12, 1908. Official duties, however, may prevent my presiding at the initial meeting of the Congress, in which case I will deputize Secretary Cortelyou.

The importance of the crusade against tuberculosis, in the interest of which this Congress convenes, cannot be overestimated when it is realized that tuberculosis costs our country two hundred thousand lives a year, and the entire world a million lives a year, besides constituting a most serious handicap to material progress, prosperity and happiness, and geing an enormous expense to society, most often in this walks of life where the burden is least bearable.

Science has demonstrated that this disease can be stamped out, but the rapidity and completeness with which this can be accomplished depends upon the promptness with which the new doctrines about tuberculosis can be inculcated into the minds of the people and engrafted upon our customs, habits and laws. The presence in our midst of representatives of world wide workers in this magnificent cause gives an unusual opportunity for accelerating the educational part of the program.

The modern crusade against tuberculosis brings hope and bright prospects or recovery to hundreds and thousands of victims of the disease, who under old teachings were abandoned to despair. The work of this Congress will bring the results of the latest studies and investigations before the profession at large and place in the hands of our physicions all the newest and most approved methods of treating the disease—a knowledge which will add many years of valuable life to our people and will thereby increase our public wealth and happiness.

The International Congress on Tuberculosis is in the interest of universal peace. By joining in such a warfare against a common foe the peoples of the world are brought closer together and made to better realize the brotherhood of man; for a united interest against a common foe fosters universal friendship. Our country which is honored this year as the host of other nations in this great gathering of leaders and experts and as the custodian of the magnificent exhibit which will be set up by the entire world, should manifest its appreciation by giving the Congress a setting worthy of the cause, of our guests, and of ourselves. We should endeavor to make it the greatest and most fruitful Congress which has yet been held, and I assure you of my interest and services to that end.

With expressions of appreciation for the compliment conferred in extending the invitation to become president of the Congress.

Very respectfully,

THEODORE ROOSEVELT.

